# Understanding development of jugular bulb stenosis in vein of galen malformations: identifying metrics of complex flow dynamics in the cerebral venous vasculature of infants

**DOI:** 10.3389/fphys.2023.1113034

**Published:** 2023-05-10

**Authors:** Sara Hadad, Shivani D. Rangwala, Jeffrey N. Stout, Fernando Mut, Darren B. Orbach, Juan R. Cebral, Alfred P. See

**Affiliations:** ^1^ Department of Bioengineering, George Mason University, Fairfax, VA, United States; ^2^ Cerebrovascular Surgery and Interventions Center, Department of Neurosurgery, Boston Children’s Hospital, Boston, MA, United States; ^3^ Department of Neurosurgery, University of Southern California LAC+USC, Los Angeles, CA, United States; ^4^ Division of Newborn Medicine, Boston Children’s Hospital, Boston, MA, United States; ^5^ Neurointerventional Radiology, Boston Children’s Hospital, Boston, MA, United States

**Keywords:** vein of galen malformation, magnetic resonance imaging, cerebral angiography, blood flow velocity, jugular foramen, cerebrovascular circulation, fluid dynamics

## Abstract

**Introduction:** Computational fluid dynamics (CFD) assess biological systems based on specific boundary conditions. We propose modeling more advanced hemodynamic metrics, such as core line length (CL) and critical points which characterize complexity of flow in the context of cerebral vasculature, and specifically cerebral veins during the physiologically evolving early neonatal state of vein of Galen malformations (VOGM). CFD has not been applied to the study of arteriovenous shunting in Vein of Galen Malformations but could help illustrate the pathophysiology of this malformation.

**Methods:** Three neonatal patients with VOGM at Boston Children’s Hospital met inclusion criteria for this study. Structural MRI data was segmented to generate a mesh of the VOGM and venous outflow. Boundary condition flow velocity was derived from PC-MR sequences with arterial and venous dual velocity encoding. The mesh and boundary conditions were applied to model the cerebral venous flow. We computed flow variables including mean wall shear stress (WSSmean), mean OSI, CL, and the mean number of critical points (nCrPointsmean) for each patient specific model. A critical point is defined as the location where the shear stress vector field is zero (stationary point) and can be used to describe complexity of flow.

**Results:** The division of flow into the left and right venous outflow was comparable between PC-MR and CFD modeling. A high complexity recirculating flow pattern observed on PC-MR was also identified on CFD modeling. Regions of similar WSSmean and OSImean (<1.3 fold) in the left and right venous outflow channels of a single patient have several-fold magnitude difference in higher order hemodynamic metrics (> 3.3 fold CL, > 1.7 fold nCrPointsmean). Specifically, the side which developed JBS in each model had greater nCrPointsmean compared to the jugular bulb with no stenosis (VOGM1: 4.49 vs. 2.53, VOGM2: 1.94 vs. 0, VOGM3: 1 vs. 0). Biologically, these regions had subsequently divergent development, with increased complexity of flow associating with venous stenosis.

**Discussion:** Advanced metrics of flow complexity identified in computational models may reflect observed flow phenomena not fully characterized by primary or secondary hemodynamic parameters. These advanced metrics may indicate physiological states that impact development of jugular bulb stenosis in VOGM.

## Introduction

Vein of Galen malformations (VOGM) are arteriovenous shunts which arise between the choroidal arterial system, and other local recruited arterial supply, and the persistent embryonic precursor to the vein of Galen, the median prosencephalic vein of Markowski (Saliou et al., 2016; Berenstein and Niimi; Berenstein et al., 2019). These patients may present with high output cardiac failure as a neonate due to high flow shunt. Other clinical manifestations include hydrocephalus and enlarging head circumference, seizures, encephalomalacia and developmental delay (Berenstein and Niimi; Hoffman et al., 1982; Johnston et al., 1987; Lasjaunias et al., 1989a; Lasjaunias et al., 1989b). The high flow nature of the fistula in VOGM has an unpredictable natural history and classically, there is no development of a deep venous system. Further, there is remodeling of the venous system in response to arteriovenous shunting of the malformation. One feature of VOGMs that remains under investigation is jugular bulb stenosis (JBS), which rarely is present at birth but can develop in some cases as the child develops and undergoes treatment (Saliou et al., 2016). JBS etiology is not well understood and the development of JBS may compromise venous outflow, causing complications related to venous hypertension. Various theories suggest the development of JBS in VOGM to be from intrinsic vessel factors or extrinsic factors (Lasjaunias et al., 2006; Saliou et al., 2016; Agarwal et al., 2017; Gupta et al., 2021; Sundararajan et al., 2021). Several studies have retrospectively reviewed patients who develop JBS in VOGM to understand risk factors and clinical implications (Lasjaunias et al., 2006; Saliou et al., 2016). Similar to the development of venous stenosis in the setting of flow disturbances in peripheral arteriovenous fistulas (AVFs) (Jia et al., 2015; Yang et al., 2020) or venous outflow stenosis in arteriovenous malformations (AVMs) of the brain (Alqadi et al., 2019), we hypothesize the development of JBS in VOGM is due to complex hemodynamic changes at focal points along the malformation.

Among computational simulations, image-based computational fluid dynamics (CFD) has become a favorable way to study cerebrovascular pathology, primarily aneurysms (Sforza et al., 2009; Hadad et al., 2021) and stroke (Rahma et al., 2022). Similarly, 4D flow magnetic resonance imaging (MRI) has been used to assess the blood flow over a cardiac cycle within both arteries and veins and can complement CFD analysis (Hope et al., 2009; Sforza et al., 2009; Schnell et al., 2017; Alqadi et al., 2019; Hadad et al., 2021; Rahma et al., 2022). CFD and 4D flow MRI technology application to VOGM offers a quantitative assessment of hemodynamic patterns within the malformation. There is no prior study to demonstrate the role of CFD in the underlying mechanism of JBS in patients with VOGM. In this study, we aim to develop patient-specific models for infants with VOGM to investigate the differences in flow patterns which lead to development of jugular bulb stenosis. We hypothesize that there are changes in the quality of flow in areas of venous tortuosity, such as the jugular bulb, which result in the development of JBS in patients with VOGM.

## Methods

**FIGURE 1:**
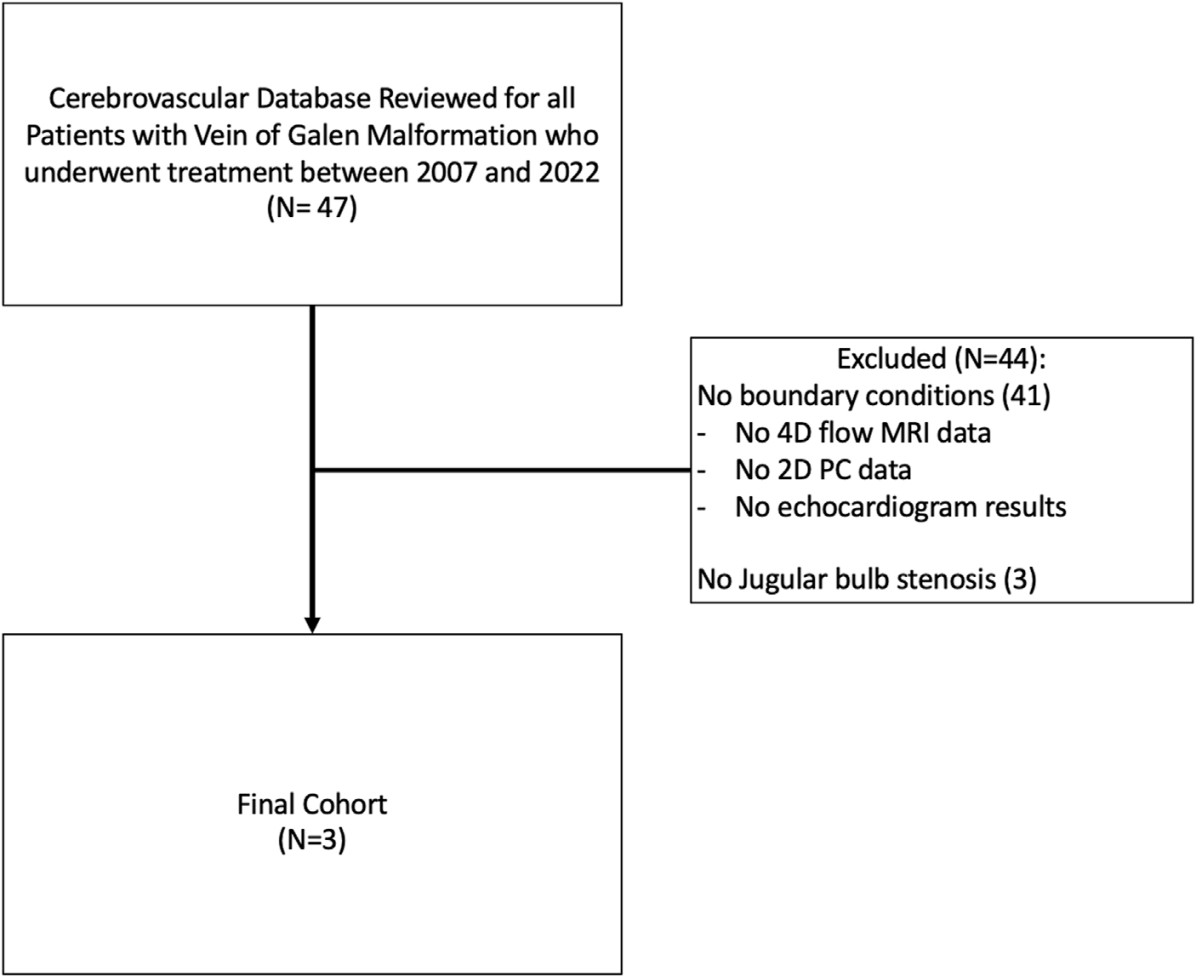
Inclusion/Exclusion criteria for study. Our institutional database of vascular patients was reviewed for all patients with Vein of Galen Malformation who underwent treatment between 2007 and 2022. Patients were excluded if they had no jugular bulb stenosis on subsequent imaging or if there was inadequate data to perform CFD analysis (either baseline comprehensive echocardiogram data of cardiac output or 4D flow MRI for flow within the malformation).

A prospectively collected vascular database at Boston Children’s Hospital was retrospectively reviewed for Vein of Galen malformations between 2007 and 2022. Patients who developed jugular bulb stenosis (JBS) and had 4D flow MRI data/or echocardiogram data (to determine boundary conditions) available were included. Patients with incomplete radiographic imaging or incomplete boundary condition data were excluded. Patients who did not undergo treatment or who died were excluded. In patients without flow MRI data, such as VOGM1, early echocardiogram results were reviewed for baseline hemodynamic boundary parameters. Clinical and radiographic data was reviewed by the neurosurgical team members (SDR, APS), flow MRI acquisition and analysis was completed by our MR physicist (JS), and CFD analysis was completed by our collaborators (SH, JC). Study inclusion methodology is further described in Figure 1.

### Patient population and procedural details

Three patients were identified who met inclusion criteria for this study. All patients were diagnosed prenatally and followed closely at birth with no concerning features of hemodynamic or neurological compromise. As per institutional policy, we closely followed each patient with MRI/MRA imaging and subsequent head ultrasounds every 2–4 weeks and if the ventricles and malformation were stable, we would conservatively manage until the patient was older for planned, staged embolization. If there were signs of clinical decompensation (cardiac failure not responsive to medical management, ventriculomegaly and elevated intracranial pressures, seizures, neurological injury) we would intervene earlier. A summary of clinical features is shown in Table 1.

**TABLE 1:** General clinical information and JBS natural history in 3 VOGM patients.

Case	Diagnosis	Clinical status at birth	Delayed symptoms or radiographic findings?	Age at first embolization (mo)	Age at development of JBS (mo)	JBS laterality	Area of JB on follow-up imaging (L, R mm^2^)	Age at initial model	4D flow MRI available?
VOGM 1	Prenatal	Stable, no heart failure or hydrocephalus	N/A	10	4	Left	32.4, 97.7	1 day	No
VOGM 2	Prenatal	Stable, mild heart failure managed medically	Seizures at 1 month	2.5	2	Left	15.6, 31.4	1 day	Yes
VOGM 3	Prenatal	Stable, no heart failure or hydrocephalus	Ventriculomegaly at 6 months	7	6	Right	60.8, 44.9	1 day	Yes

**FIGURE 2:**
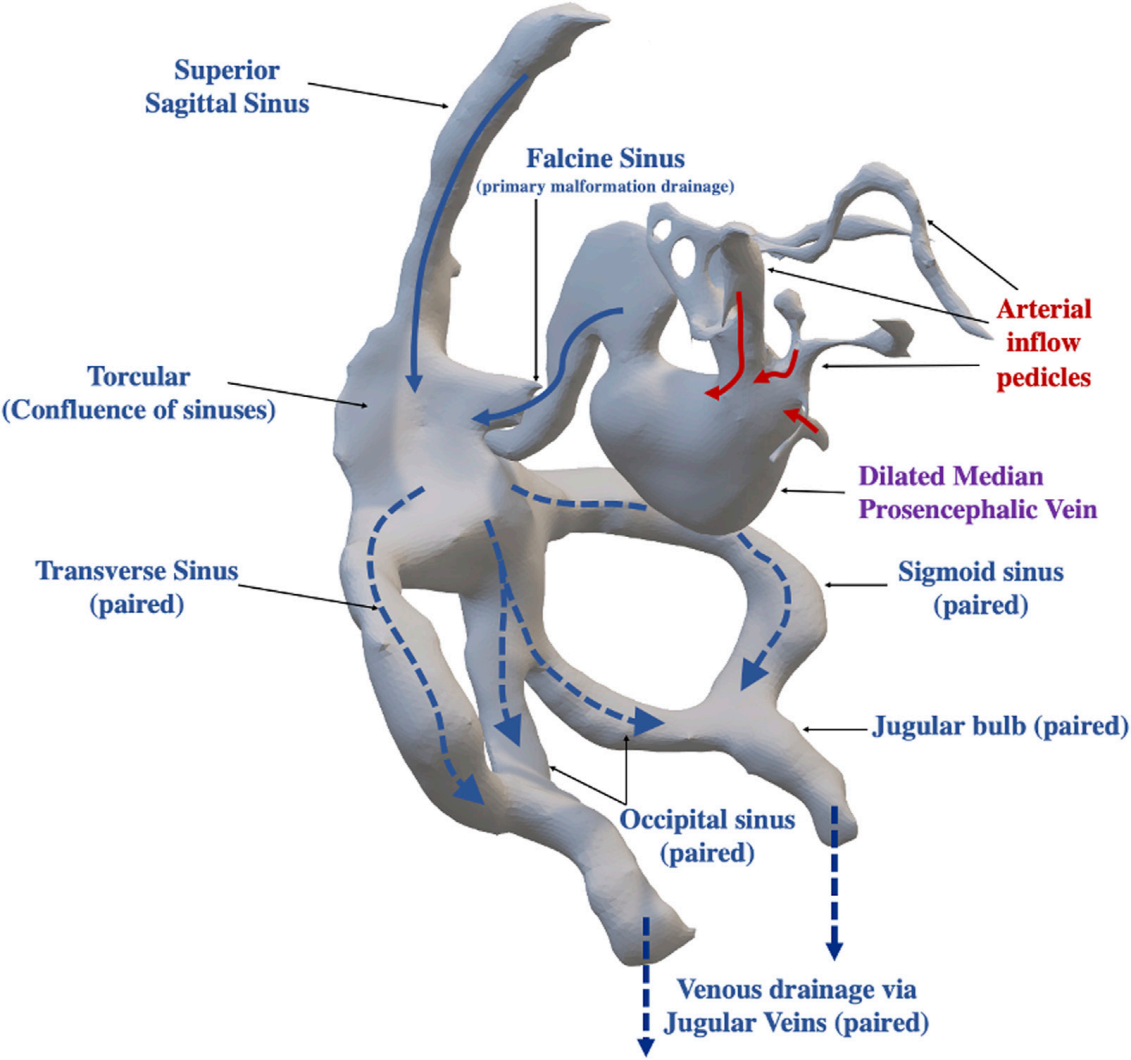
Overview of Vein of Galen Arteriovenous Anatomy and Arteriovenous Flow Pattern within the Malformation. Arteriovenous shunting is central to the anatomic structure of the vein of Galen malformation, with arterial feeders (red arrows) directly draining into a dilated median prosencephalic vein (precursor to the Vein of Galen Malformation). The primary outflow of the dilated median prosencephalic vein is the falcine sinus which drains into the confluence of sinuses (Torcular), which receives venous drainage from the superior sagittal sinus. The torcular then divides into several major outflow sinuses (dashed arrows), including the right and left transverse sinuses and sigmoid sinuses. In some infants, there is additional outflow via the occipital sinuses which then merge with the sigmoid sinus proximal to the jugular bulb. Beyond the jugular bulb the sinuses exit the skull as the jugular veins (paired structure).

All patients were taken for diagnostic cerebral angiogram with planned embolization for target feeders. Per our institutional policy, patients were intubated and under general anesthesia for the duration of the procedure. Given contrast limitations in small children, a full diagnostic angiogram was deferred until after all target feeders could be embolized. The usual strategy was transarterial embolization with a combination of coils, N-butyl-2-cyanoacrylate (nBCA) or Onyx™. Once target feeders were embolized or flow within the malformation was reduced sufficiently or the total contrast allotment for the study was completed, the procedure was concluded. At our institution, we obtain an immediate post operative MRI/MRA if there is concern for significant flow reduction and possible thrombosis formation within the malformation, otherwise additional imaging is usually deferred for 3–6 months if the patient remains clinically well. An overview of the standard anatomy and typical pattern of blood flow with arteriovenous shunting in a VOGM is described in Figure 2.

All imaging is reviewed by neurosurgeons (SDR, APS) and neurointerventionalist (DBO) with the MR physicist (JNS), who initially correlate 4DMRI results to clinical results. All relevant imaging and angiographic runs are reviewed to assess flow differences between various anatomic structures to assess if the MR flow quantitative values correlated to real time angiography of the specific patient. This correlation is done before application of the BC to CFD modeling.

### Computational modeling

**FIGURE 3:**
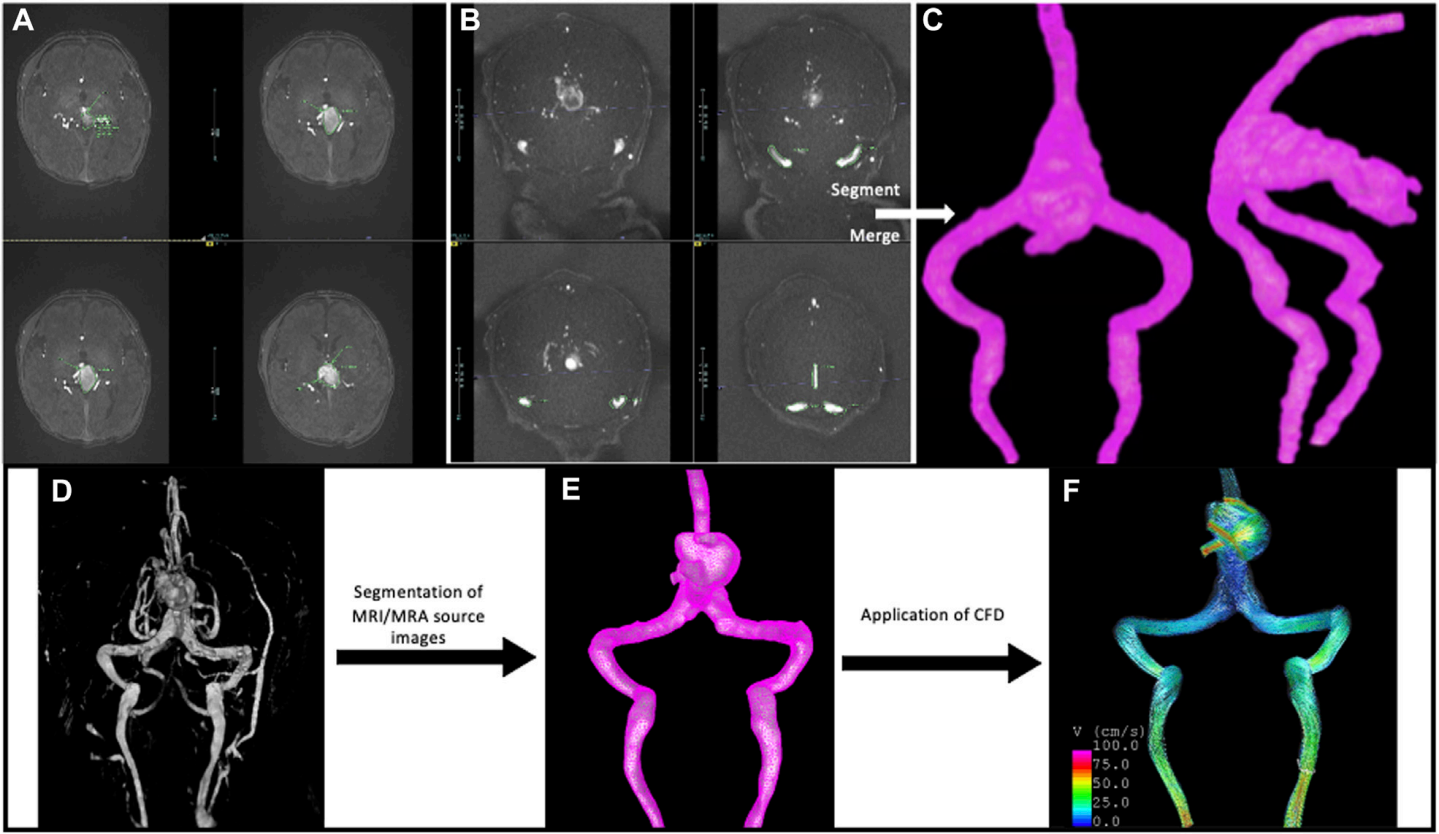
Overview of workflow using **(A)** time of flight (TOF) magnetic resonance angiography and **(B)** TOF magnetic resonance venography to segment the **(C)** Vein of Galen model. MRA and MRV data is segmented separately and then merged to create the entire structure of the VOGM and the venous drainage to the level of the jugular bulb. This anatomy would otherwise not be captured within one sequence alone. The patient specific imaging data is also used to create **(D)** MRI volume rendered 3D structure and **(E)** mesh of VOGM with ultimate **(F)** application of CFD for analysis of flow dynamics within the malformation.

**FIGURE 4:**
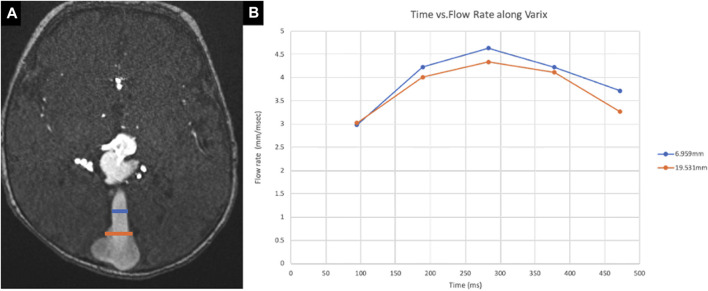
Measurement by 4D flow MRI along the falcine sinus. **(A)** The flow rate at different sites (6.9 mm and 19.5 mm) along the falcine sinus (pathologic flow) of VOGM2 has been measured by 4D flow MRI, as shown by the blue and orange annotations on axial 2D TOF MRA. **(B)** The inflow rates at these two locations are plotted over a sampled cardiac cycle. The average inflow of the two locations has been used as the inflow boundary condition in CFD simulations.

**FIGURE 5:**
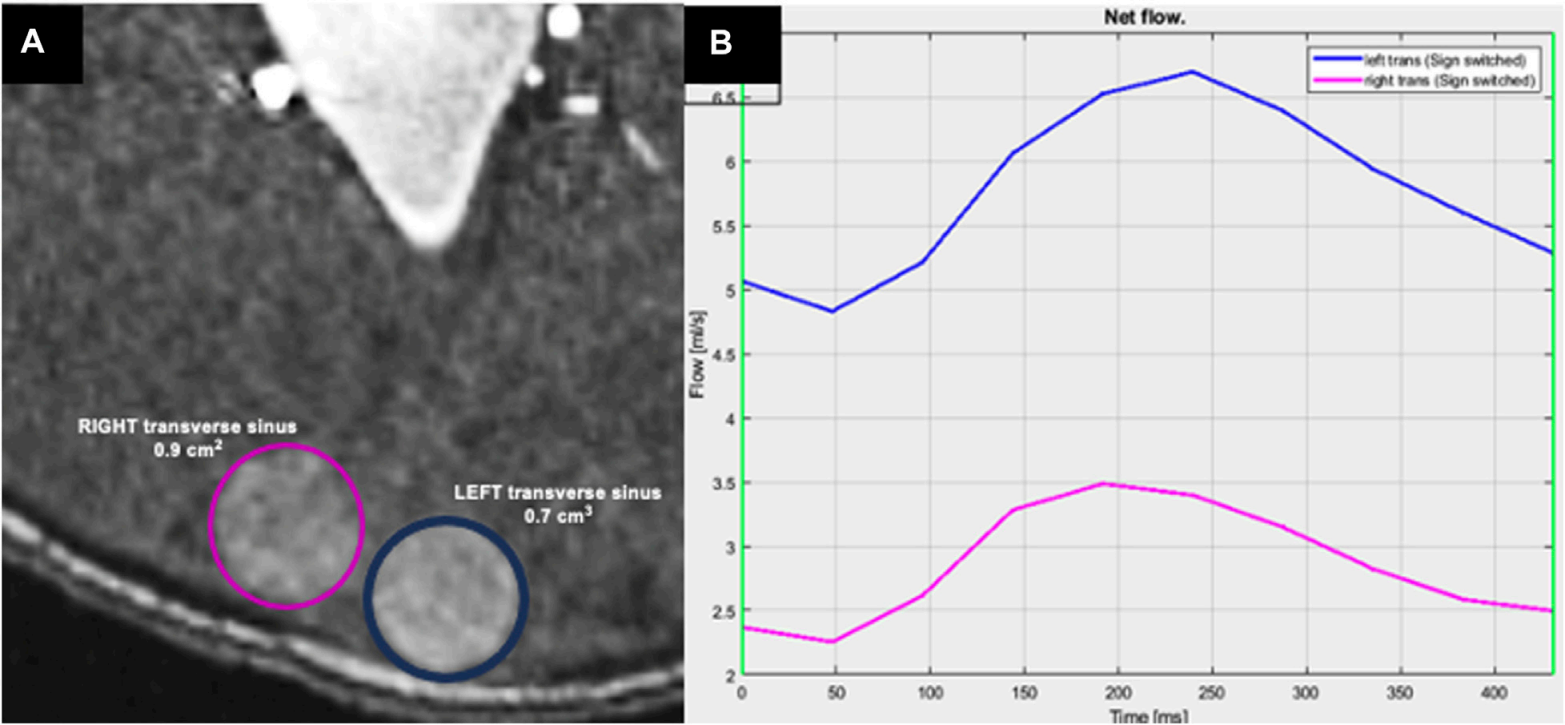
Measurement of flow rate within the transverse sinuses by 2D PCMRI. **(A)** Axial TOF MRI showing the location of measurements with color corresponding to **(B)** the plot showing the flow rate at the left (blue) and right (pink) transverse sinuses (division downstream to the confluence of sinuses) as measured by 2DPCMRI. Plot in **(B)** shows the flow rate over a sampled cardiac cycle, with a higher flow rate through the left transverse sinus at this segment.

**FIGURE 6:**
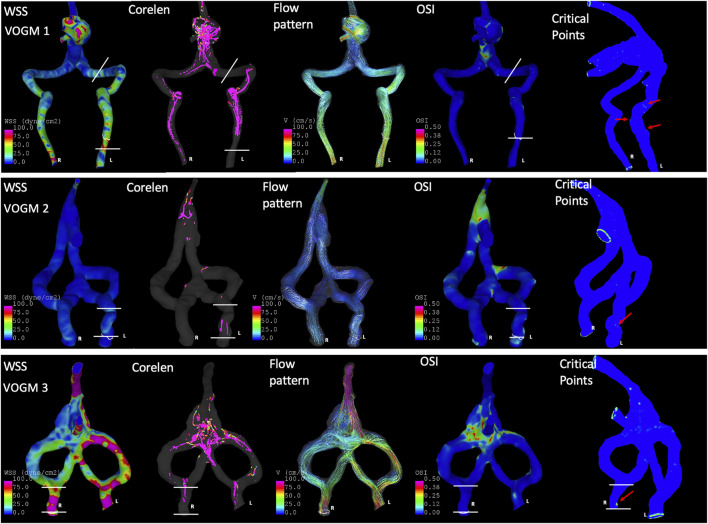
Application of CFD to assess hemodynamics for VOGM before the development of JBS. Laterality is indicated in the first column as patient R or L. VOGM1 demonstrated symmetric wall shear stress (First Column, WSS) but increased flow complexity (Second column, core line length, i.e., Corelen) and increased mean number of critical points on the side which ultimately develops JBS on subsequent modeling (Fifth column, Critical Points indicated by red arrows). In the first column of WSS maps–the color scale is identical on all models—0–100 dyne/cm^2^ which ranges from blue to pink, respectively). VOGM2 demonstrates comparable WSS bilaterally but slightly increased CoreLen (second column), OSImean [fourth column, Oscillatory Shear Index (OSI) mean, averaged across the jugular bulb] and number Critical Points (Fifth column, red arrows) on the side which develops stenosis. In VOGM3, the key difference between sides is the Critical points (Fifth column, red arrow) which is a smaller difference compared to the other two models, but this patient develops only mild stenosis on this side eventually. The spatial area across which averages are determined is described by the white lines for each VOGM model.

MR images were used for 3D vascular segmentation to create patient-specific CFD models of the VOGM and jugular bulb using previously described methods (Cebral et al., 2005) and this workflow is depicted in Figure 3. Specifically, the MRI protocol details for each patient model is provided in Supplementary Table S1. The TOF imaging was utilized for segmentation of the venous varix and any large arterial feeders, and the post contrast MRI sequences were utilized for segmentation of the venous drainage network of the malformation. There was minimal compromise in quality of imaging due to flow changes for segmentation of the venous system because the structures are significantly large (5 mm or greater). However, it should be noted that segmentation of smaller arterial feeders was not done in the study of JBS, and this anatomic area of segmentation could be compromised by flow signal artifact on TOF. Navier-Stokes equations were solved numerically to perform the computational fluid dynamics (CFD) simulations. Pulsatile flow conditions were derived from echocardiogram (Table 2) or 4D flow or 2D PC MRI results prior to treatment (Figures 4, 5). Pressure zero was applied for the outlet boundary conditions for both jugular veins. Vessel walls were rigid and no-slip boundary conditions were applied to the vascular walls. CFD simulations were done for two cardiac cycles with a time-step of 0.01 s. The results of the second cycle were used to characterize the local hemodynamics of the jugular bulb sinuses. An example of local hemodynamics of each VOGM case is illustrated in Figure 6. Additional information demonstrating CFD simulations over one cardiac cycle for VOGM1 are shown in Supplementary Videos S1A–D.

**TABLE 2:** Demonstrates boundary condition calculations for VOGM1 from early ECHO results. These are for the patient at 4.5 months of age, prior to intervention. Values are calculated as shown and reference numbers are per mean based on body surface area.

Height 67.5 cm
Weight 7.2 kg
Body surface area (BSA) 0.37 m^2^
Heart rate 138 bpm (mean for age 108 bpm)
2D left ventricular end diastolic volume (2D LV EDV) 24.27 mL (mean for BSA 18.28 mL)
2D left ventricular end systolic volume (2D LV ESV) 9.43 mL (mean for BSA 6 mL)
Stroke volume = EDV—ESV
24.27 mL–9.43 mL = 14.84 mL
Cardiac output (CO) = Heart rate × Stroke volume
138 bpm × 14.48 mL = 2047.92 mL/min
Compared to BSA reference mean calculated CO of 1,334.10, there is 713.82 mL/min of excess flow presumed to be supplying the malformation

### Flow MRI acquisition and processing

Flow MRI was used to measure intracranial hemodynamics. 4D flow MRI was prescribed as a slab centered on the Circle of Willis, and obliqued to capture flow information in the main venous varix and falcine sinus (repetition time (TR) = 82.6–92.8 ms, echo time (TE) = 3–3.2 ms, resolution = 1 mm isotropic, field of view = 220 mm, encoding velocity (VENC): 80–160 cm/s, k-t acceleration R = 5, 5 cardiac phases) (Schnell et al., 2017). Large venous structures would occasionally extend beyond the slab and so 2DPCMRI was used to measure flow in those targets (TR = 65.76 ms, TE = 5.02 ms, resolution = 0.8 × 0.8 × 4 mm^3^, field of view = 240 mm, VENC = 50–100 cm/s through plane, 10 cardiac phases). For 2DPCMRI, vessel regions of interest were manually defined in Segment (Heiberg et al., 2010) to calculate flows. 4D flow data was corrected for Maxwell terms during reconstruction, and for eddy currents and velocity noise (Bernstein et al., 1998; Bock et al., 2007). Vessel-wise flows were then calculated in a semi-automated fashion (Vali et al., 2019). Vessels for flow measurements were targeted based on patient specific features for CFD, often including superior sagittal sinus and falcine sinus flow and transverse sinuses.

### Data analysis

To characterize the hemodynamics around the jugular bulb, flow variables were computed including mean wall shear stress (WSSmean), mean oscillation of WSS (OSImean), flow pattern complexity (corelen), and mean critical points in the WSS vector field (nCrPointsmean) (Mut et al., 2011). Oscillatory shear index (OSI) measures the deviation of the wall shear stress vector with respect to the mean (time-averaged) wall shear stress. The mean quantities are spatially averaged over the region of interest, which is the jugular bulb (specifically across the sigmoid sinus into the jugular vein) in each malformation model. This area of interest excluded the occipital sinus, when present (Figure 2 for anatomical description of occipital sinus; Figure 6, white lines indicate region of interest for each model). For VOGM1 we did not have 4D flow boundary conditions given this patient treatment occurred prior to implementation of 4Dflow MRI at our institution. We utilized initial echocardiogram data, calculated as described in Table 2, to determine amount of excess flow to the malformation for this patient. As a validation technique, we applied boundary conditions determined from VOGM2 4D flow MRI to VOGM1 model to assess how different boundary conditions impact hemodynamic metrics (Table 3, *VOGM1* with 4D MRI BC*).

**TABLE 3:** Hemodynamics of VOGM with and without JBS in pre-developed patient-specific models.

Case	WSS mean		Corelen		nCrPoints mean		OSI mean	
	Stenosis	No-stenosis	Stenosis	No-stenosis	Stenosis	No-stenosis	Stenosis	No-stenosis
VOGM 1	22.27	17.48	26.48	7.86	4.49	2.53	0.003	0.0003
VOGM1 with 4DMRI BC*	8.43	6.07	12.4	1.3	4.94	2.04	0.007	0.003
VOGM 2	4.8	3.6	3.77	0.00005	1.94	0	0.012	0.005
VOGM 3	99.32	83.47	0.17	0.17	1	0	0.0003	0.007

*4DMRI BC, are determined from VOGM2 but applied to VOGM1 CFD, model for calculation of this row of hemodynamic metrics. BC, boundary conditions. WSS, wall shear stress. Corelen, length of flow core line. nCrPoints, number of critical points. OSI, oscillatory shear index.

Where the shear stress vector field is zero (stationary point) that location is mathematically called a critical point. In CFD models, linear interpolation was used to find the location of critical points within an element where the WSS vector field is smaller than 10^−5^. As Mazzi et al. (2021) have shown, there are several types of critical points such as node sources, focus sources, and saddle points that can be identified based on their local derivation around the stationary point (Arzani and Shadden, 2018). Critical points can describe the blood flow topology and the local WSS field which affects the endothelial function as the WSS vector direction, and its magnitude can change drastically in vicinity of these points. Thus, the mean number of critical points over a cardiac cycle can describe the overall flow topology and measure the number of regions with the abnormal WSS field and consequently the affected endothelium (Baek et al., 2010).

## Results

**FIGURE 7:**
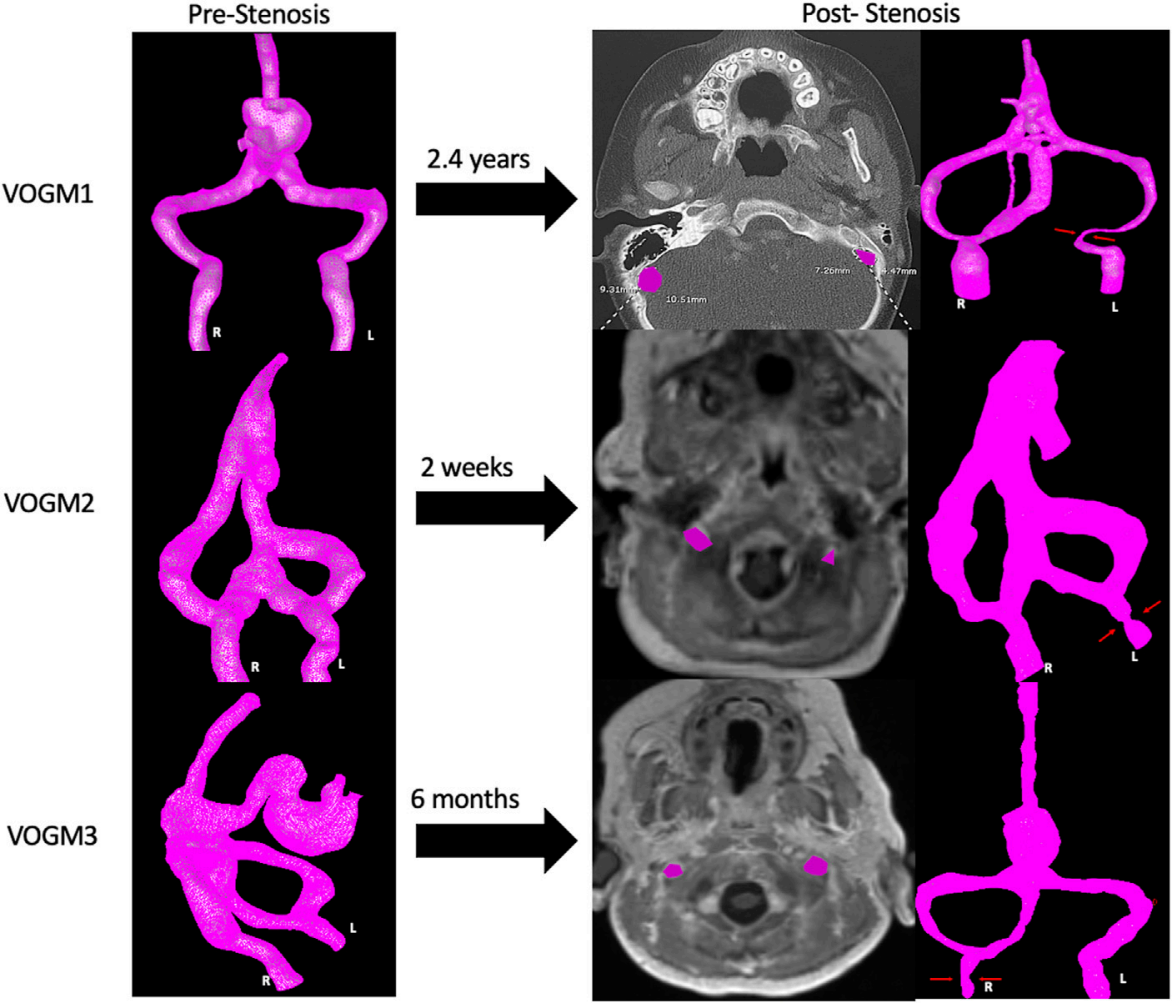
Development of Jugular Bulb Stenosis. Development of stenosis for three VOGM cases in our series, as demonstrated by a recent axial cut image at the level of the jugular bulb (JB highlighted in purple on each side) with the corresponding 3D models where the area of stenosis is indicated (red arrows).

Three cases developed JBS at one side of the jugular bulb (Table 1). Computed hemodynamic results at the jugular bulb on each side before development of stenosis are presented in Table 3. The mean WSS did not show a distinct difference on each side for any patient. However, the complexity of the flow pattern was at least 3 times larger at the jugular bulb with subsequent stenosis compared to the side without subsequent stenosis in VOGM1. In addition, the jugular bulb with subsequent stenosis has more than 1.5 times more critical points of the wall shear stress field and 2 times larger oscillatory shear index in comparison to the jugular bulb without subsequent stenosis in VOGM1 and VOGM2. An example of flow visualization is presented in Figure 6 for each patient case. The figure shows that prior to the development JBS, that side has a more complex flow and has a larger number of critical points and OSImean compared to the other side, most obvious for VOGM1. Supplementary Table S2 describes the variety of critical points observed for each VOGM model. Further demonstration of the CFD for VOGM is shown in Supplementary Videos 1A–D which illustrate the difference in higher order hemodynamic parameters (CoreLen and nCrPtmean) on the side which ultimately develops JBS. The change in cross sectional area at the jugular bulb is calculated for each patient in Table 4. Interestingly, we note a smaller difference of complexity of flow and smaller percentage of cross-sectional area reduction in VOGM3 between the side which developed stenosis and the side that did not. This finding clinically correlates to the lower flow malformation properties of VOGM3 compared to VOGM1 and VOGM2. Correlation between initial model and most recent cross-sectional imaging and 3D model demonstrating JBS is shown in Figure 7.

**TABLE 4:** Cross sectional area at the Jugular Bulb before and after development of stenosis.

Case	PRIOR to development of JBS (mm^2^)		After development of JBS (mm^2^)		% Change in cross sectional area	
	Stenosis	No-stenosis	Stenosis	No-stenosis	Stenosis	No-stenosis
VOGM1	0.3166	0.3552	0.0103	0.0893	97%	75%
VOGM2	0.5186	0.5516	0.0717	0.2280	86%	59%
VOGM3	0.0789	0.1091	0.0357	0.0927	55%	15%

JBS, jugular bulb stenosis.

## Discussion

Vein of Galen Malformations can develop jugular bulb stenosis, however the underlying pathophysiology is not well understood. This structural change can precipitate symptoms of cerebral venous hypertension, hydrocephalus or cerebral hemorrhage, each with significant neurological and neurodevelopmental impact (Lasjaunias et al., 2006; Saliou et al., 2016). Prediction of JBS development may improve interventional planning and treatment for VOGM and guide careful observation of patients who are at higher risk for clinical decline (Khullar et al., 2010).

Several studies have used image-based CFD models to assess the severity of arterial stenosis and study the local hemodynamics of arterial stenosis (Schirmer and Malek, 2012; Liu et al., 2017; Albadawi et al., 2021). Venous stenosis development has been investigated with the application of CFD in certain vascular pathophysiology. Specifically looking at arteriovenous fistulas or veins subjected to high arterial pressures with subsequent development of venous stenosis, Yang et al. (2020) have shown the effect of hemodynamics parameters and flow disturbance on endothelial cells (Chien, 2008) and its thickening (Dobrin, 1995). Additionally, recent advances with 4D flow MRI allow for quantitative assessment of intracranial vascular hemodynamics, complementing results from CFD (Castle-Kirszbaum et al., 2020; Misaki et al., 2021). This study is the first to focus on the application of CFD and flow MRI results to predict the development of JBS in patients with VOGM.

Our results indicated that the flow complexity, number of critical points, and the oscillation of WSS are greater in the jugular sinus which will develop stenosis compared to the other side which will not develop stenosis. Several factors affect endothelial function, including the magnitude and direction of wall shear stress (Cheng et al., 2019). In the current study, although the magnitude of WSS is similar between bilateral jugular bulbs in an initial state CFD simulation, the oscillation of WSS is larger at the side that subsequently develops stenosis. In addition, Chen et al. (2017) illustrate a correlation between higher OSI and thickened inner vascular walls. Stenosis is a localized phenomenon, and the identified flow conditions in this study are favorable conditions for deforming the vascular wall and developing stenosis. The association of critical points in the cerebral venous system or jugular bulb stenosis has not been previously reported.

Our study is the first largest patient cohort application of CFD to pediatric VOGM, and the first to assess complementary flow MRI. A previous study by Hassan et al. demonstrated feasibility of CFD analysis in treatment planning for a single VOGM discovered in a 22 year old (Hassan et al., 2003). However, this case study only modeled the arterial anatomy in the feeder within a focused geometric region with a similar technique to modeling of adult arterial aneurysms. The modeling of JBS pathophysiology extends over half the cranial space, involving more risk of propagated computational error, and applies patient-specific time-resolved volumetric boundary conditions. As a proof of concept with favorable results, there is a promising future to CFD and flow MRI application in VOGM, and more broadly in larger scale venous modeling that differs from focused, smaller scale arterial modeling, as in the case of cerebral aneurysms. This is advantageous primarily because of the medical comorbidities and patient weight limitations that prevent most infants with a VOGM from obtaining complete diagnostic angiograms to minimize contrast and radiation associated risks. Non-invasive MRI and CFD applications allow for assessment of flow to predict high risk features of VOGM, such as JBS, prior to occurrence and can help guide management in this patient population.

Our study has several limitations. One is the small number of cases in which we have demonstrated CFD feasibility and flow MRI analysis. The small patient cohort may limit the generalizable nature of our results and validity of our study. Specifically, we did not do additional validation experiments to compare 4D flow and 2D flow MRI data where they coincided for specific patients as this has already been established in the literature (Aristova et al., 2019). Future research involving more patients can be done to support the conclusions made in this study. Additionally, not all patients underwent a computed tomography (CT) scan after development of JBS to assess caliber of the jugular foramen (Saliou et al., 2016) which could either be contributory to development of stenosis; however, the changes in flow complexity on vascular wall are a greater predictor as they were noted to occur prior to the development of JBS. The findings of this study are based on the assumption that the CFD simulation terms used for brain aneurysms (Cebral et al., 2005; Sforza et al., 2009) are applicable to jugular vein as well. Hence, the validity of the results is dependent on this assumption and this is a potential limitation to our initial models. Future studies involving analysis of endothelial tissue samples in VOGM patients could be studied to better understand endothelial response to increased flow complexity.

## Conclusion

Hemodynamic features from subject-specific CFD models of early VOGM angioarchitecture associates with lateralizing jugular bulb stenosis. Our study demonstrated that higher order measures of flow complexity, such as average OSI, number of critical points, and core line length, are larger in the sinus prior to JBS development although WSS, as a first order hemodynamic metric, may be similar. Prediction of JBS occurrence in VOGM patients can help physicians better manage and treat this complex patient population.

## Data Availability

The raw data supporting the conclusion of this article will be made available by the authors, without undue reservation.
