# Supplementary material for: Understanding development of jugular bulb stenosis in vein of galen malformations: identifying metrics of complex flow dynamics in the cerebral venous vasculature of infants
Source: Front Physiol. 2023 May 10;14:1113034. doi: 10.3389/fphys.2023.1113034 (PMC10236198; doi:10.3389/fphys.2023.1113034)

Supplementary Materials

Supplementary Table 1. MRI protocol for MR imaging of each segmentation model.

Patient	**Model**	**Imaging Protocol**
**VOGM1**	Model 1	TOF MRA: TR=21 ms, TE=3.69ms, Resolution=0.52x0.52x0.60 mm^3 FOV133x199 mm^2<br>TOF MRV: TR=22 ms TE=5.24ms, Resolution=0.625x0.625x2 mm^3 FOV=123x160 mm^2
	Model 2	CT protocol: Resolution=0.4x0.4x3 mm^3
**VOGM2**	Model 1	TOF MRA: TR=20ms, TE=3.19m, Resolution=0.5x0.5x0.5 mm^3 FOV= 70.5 mm^2<br>T1-weighted post-contrast MPRAGE : TR=1880ms TE=3.02ms, Resolution=0.8 x 0.8 x 0.9 mm^3, FOV = 90x90 mm^2
	Model 2	TOF MRA: TR=24 ms, TE =4.05 ms, Resolution=0.5x0.5x0.6 mm^3, FOV = 126x140 mm^2<br>T1-weighted post-contrast MPRAGE: TR=2080 ms TE=2.43ms, Resolution=0.85 x0.85 x0.90 mm^3, FOV =140x140 mm^2
**VOGM3**	Model 1	TOF MRA: TR=24 ms TE=3.69 ms, Resolution=0.5x0.5x 0.6 mm^3 FOV = 189 x 199 mm^2<br>T1-weighted post-contrast MPRAGE: TR=2100ms TE=2.57ms, Resolution=0.7x0.7x0.9mm^3 FOV = 256 x 256 mm^2
	Model 2	TOF MRA: TR=21ms, TE=3.69 ms Resolution = 0.52x0.52x0.7 mm^3, FOV=181x199 mm^2<br>T1-weighted post-contrast MPRAGE: TR=2000 ms TE=2.45ms Resolution= 0.86x0.86x0.90mm^3, FOV=179x179 mm^2

Abbreviations

(TR) Repetition time
(TE) Echo Time
(FOV) Field of View
(TOF) Time of flight
(MRA) Magnetic Resonance Angiogram
(MRA) Magnetic Resonance Venogram

Supplementary Table 2. Average number and type of critical points over a cardiac cycle for each VOGM model.

**Case name**	**Stenosis status**	**numSADDLE**	**numNODE _SOURCE**	**numNODE_ SINK**	**numFOCUS _SOURCE**	**numFOCUS _SINK**
**VOGM1**	stenosis	1	1	0.58	0.91	1
	no-stenosis	1	0	0.66	0	0.87
**VOGM2**	stenosis	0.82	0.27	0.85	0	0
	no-stenosis	0	0	0	0	0
**VOGM3**	stenosis	1	0	0	0	0
	no-stenosis	0	0	0	0	0

Supplementary Table 3. Inflow rates determined by 4D flow MRI as Boundary Conditions for CFD. The flow rate, averaged over a single cardiac cycle, as measured by 4D flow along the venous varix or draining falcine sinus. This region of interest correlates to the "Falcine Sinus" in Figure 2 in the main manuscript which overviews VOGM anatomy.

Case	Varix/Falcine Sinus Flow (ml/s, averaged over single cardiac cycle)
VOGM2	5.7
VOGM3	2.7

Supplementary Table 4. Calculation of Flow Conservation Error
Flow conservation error was calculated as |1- sum(transverse sinuses)/ sum(SSS,varix). Of note, the results for calculations in the first scan combine measurements from 2D and 4D flow MRI estimates, and this higher flow conservation error could be attributed to the mix of modalities. In comparison to the exclusively 4D flow MRI based observations in phantoms, this error is high for the first scan, but the second is within the range observed by Aristova et al.

	First Scan (ml/s)	Second scan (ml/s)
SSS	0.24	0.88
Varix	5.76	3.91
Right Transverse Sinus	2.85	1.97
Left Transverse Sinus	5.76	3.6
**Flow conservation error**	**44%**	**16%**

Supplementary Video 1A-D: Simulation of CFD modeling for VOGM . A. Wall Sheer Stress (WSS) B. Flow Pattern, described as velocity cm/s, C. Core line length and D. Critical Points. Overall, the simulations demonstrate the greater degree of core line length and number of critical points on the patient's left side (red circle), which is the site of ultimate JBS development.

Supplementary Figure 1: Comparison of modeled flow conditions with measured flow conditions
To compare the mean linear velocity of the right transverse sinus measured by 2D flow MRI with the mean linear velocity of the same venous sinus modeled by CFD, a skeleton has been generated along the 3D model and three points along the right transverse sinus (red rectangle) have been selected as illustrated in panel B. Panel A plots linear velocity over a cardiac cycle. The spatially averaged modeled velocity from the three points is plotted over a simulated cardiac cycle (turquoise dashed line). The mean linear velocity across that segment of the sinus as measured by 2D flow MRI is plotted in red dots. Since this is a measurement of laminar flow, there is spatial variation in measured linear velocity over the cross section; this is illustrated in this plot as the spread at each time point in a sampled cardiac cycle. The two analysis methods demonstrate comparable velocity in the same model.

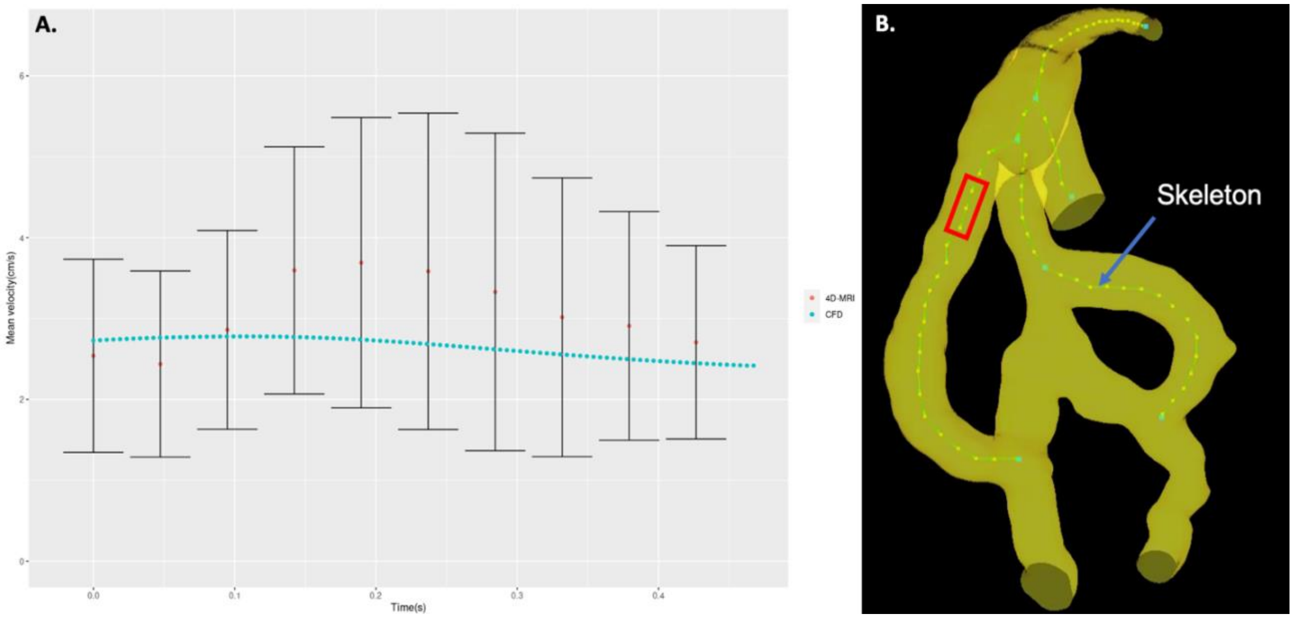

Supplementary Figure 2: Comparison of velocity profiles along the transverse sinuses between CFD and 2DPC MRI. **A**. 3D segmentation model of VOGM case 2 which demonstrates the pre-stenosis model and the slice location along the 3D structure which provided **B**. the 2D color contour velocity plot within the right (R) and left (L) transverse sinuses generated by CFD and shown on a similar cut on **C**. MRI by the 2DPC velocity profile, there is greater velocity profiles in the left transverse sinus compared to the right. Additionally, when compared to angiography imaging, there is preferential flow from the falcine sinus (higher velocity profile) into the left transverse sinus, whereas the right transverse sinus predominantly receives flow from the superior sagittal sinus (lower velocity profile). The comparison of these two side by side 2D color contour velocity plots demonstrate the correlation in results between CFD and MRI in our study.

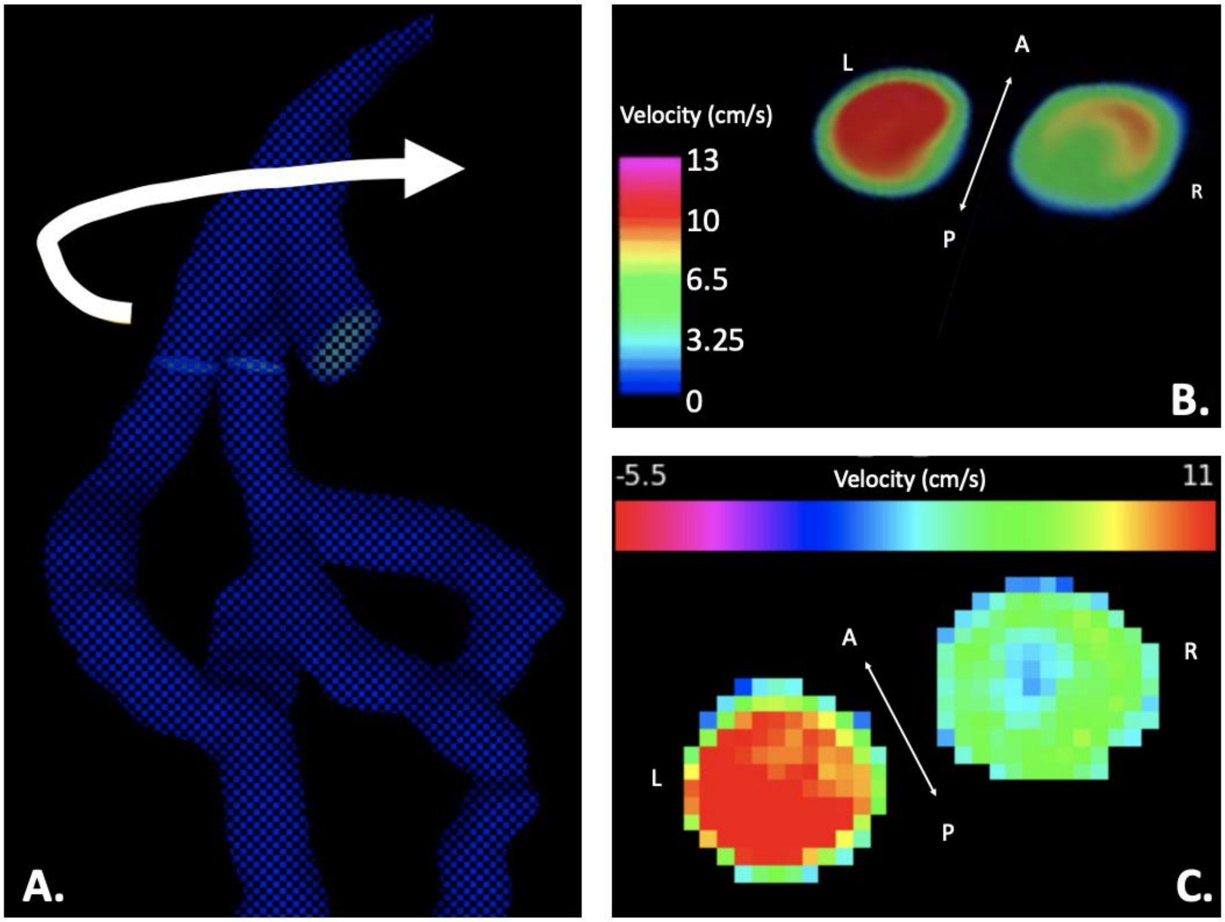

Supplement: Supplementary file 1 [file DataSheet1.PDF]
